# The Pupillary Response of the Common Octopus (*Octopus vulgaris*)

**DOI:** 10.3389/fphys.2020.01112

**Published:** 2020-09-18

**Authors:** Cecilia Soto, Almut Kelber, Frederike D. Hanke

**Affiliations:** ^1^Sensory and Cognitive Ecology, Institute for Biosciences, University of Rostock, Rostock, Germany; ^2^Vision Group, Department of Biology, Lund University, Lund, Sweden; ^3^Neuroethology, Institute for Biosciences, University of Rostock, Rostock, Germany

**Keywords:** pupil, vision, pupil light reaction, pupil dark reaction, shadow effect

## Abstract

Cephalopods have very conspicuous eyes that are often compared to fish eyes. However, in contrast to many fish, the eyes of cephalopods possess mobile pupils. To increase the knowledge of pupillary and thus visual function in cephalopods, the dynamics of the pupil of one of the model species among cephalopods, the common octopus (*Octopus vulgaris*), was determined in this study. We measured pupillary area as a function of ambient luminance to document the light and dark reaction of the octopus eye. The results show that weak light (<1 cd/m^2^) is enough to cause a pupil constriction in octopus, and that the pupil reacts fast to changing light conditions. The t_50_-value defined as the time required for achieving half-maximum constriction ranged from 0.45 to 1.29 s and maximal constriction from 10 to 20% of the fully dilated pupil area, depending on the experimental condition. Axial light had a stronger influence on pupil shape than light from above, which hints at a shadow effect of the horizontal slit pupil. We observed substantial variation of the pupil area under all light conditions indicating that light-independent factors such as arousal or the need to camouflage the eye affect pupil dilation/constriction. In conclusion, the documentation of pupil dynamics provides evidence that the pupil of octopus is adapted to low ambient light levels. Nevertheless it can quickly adapt to and thus function under brighter illumination and in a very inhomogeneous light environment, an ability mediated by the dynamic pupil in combination with previously described additional processes of light/dark adaptation in octopus.

## Introduction

The cephalopods are a molluscan class that differs from other members of the phylum by a number of characters such as the anatomy of the body and the organization of the nervous system. One of the most prominent characteristics of cephalopods are their eyes (for review see Packard, 1972; Messenger, 1979; Land, 1984; Budelmann, 1994, 1996). They are large and often actively scanning the animal’s surrounding. To some extent, cephalopod eyes resemble vertebrate camera-type eyes.

**FIGURE 1:**
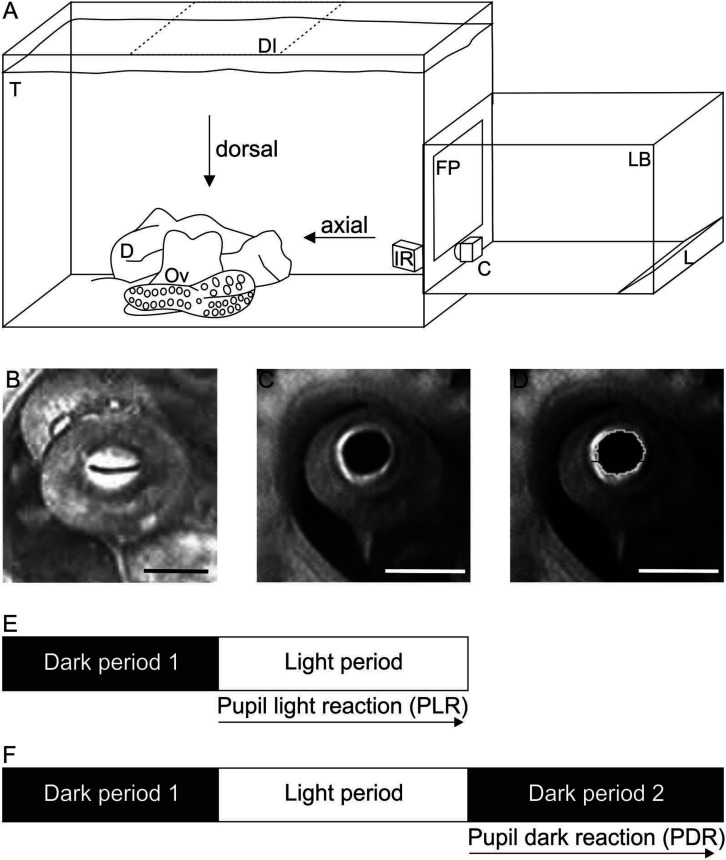
Experimental setup and procedure. **(A)** Sketch of the experimental setup to record pupillary responses. During measurements, the animal (Ov) was hiding in its den (D) in its holding tank (T). The scene was either illuminated axially with the light box (LB) and camera C as shown or from dorsal (position of light box shaded, DI; camera remains in position as during axial illumination). Within the light box, light of three reflector light bulbs (L) was reflected numerous times by aluminum foil lining the inside of the box before it indirectly hit the semitransparent front plate (FP). The scene was additionally illuminated by infrared light (IR) allowing to document the pupil in darkness/dim light conditions. Not drawn to scale. **(B–D)** Pupil of *Octopus vulgaris*. B Constricted pupil when the scene was illuminated with 170.3 cd/m^2^ axially. Absolute pupil area was 4 mm^2^ on this frame. C Dilated pupil measured in darkness under IR-light before the light was switched on. Absolute pupil area was determined as 33 mm^2^ on this frame. **(D)** Image of the dilated pupil showing how the pupil was encircled in ImageJ to determine the pupil area. Scale 10 mm. **(E,F)** Sketch of the experimental procedure to document **(E)** the pupil light reaction (PLR) and **(F)** the pupil dark reaction (PDR). For both pupil reactions, the animal was first kept in darkness (dark period 1) followed by a light period. During this light period with a specific light level set up, the pupil light reaction was documented. The pupil dark reaction was recorded in a subsequent dark period (dark period 2).

A conspicuous feature of the cephalopod eye is its pupil, which is peculiarly shaped in some species. Pupil shape varies from horizontal to U- or W-shaped in bright light depending on species (see Figure 1 and photos within for example Douglas, 2018). A number of studies have already tried to assess the function of these specific pupillary shapes (Hanlon and Messenger, 1988; Schaeffel et al., 1999; Mäthger et al., 2013; Stubbs and Stubbs, 2016) or provided descriptions of the anatomy of the iris as well as of pupillary dynamics in some species (Beer, 1897; Magnus, 1902; Wiley, 1902; Hess, 1910; Heidermanns, 1928; Weel and Thore, 1936; Froesch, 1973; Muntz, 1977; Hurley et al., 1978; Muntz and Ray, 1984; Douglas et al., 2005; Bozzano et al., 2009; McCormick and Cohen, 2012; Matsui et al., 2016).

A dynamic pupil, as present in cephalopods, generally helps (1) to balance sensitivity and resolution of an eye (Douglas, 2018), and (2) to adapt the eye to different light conditions thereby avoiding the saturation of the photoreceptors and increasing the probability of light detection. Besides pupillary changes, adaptation to light can involve the migration of screening pigment – separating the two rhabdoms of a photoreceptor and separating the distal segments of the photoreceptors, or changes in the photoreceptor length in cephalopods (Babuchin, 1864; Rawitz, 1891; Hesse, 1900; Hess, 1905; Glockauer, 1915; Hagins and Liebman, 1962; Young, 1963; Daw and Pearlman, 1974; Suzuki et al., 1985; Suzuki and Takahashi, 1988; Gleadall et al., 1993; Bozzano et al., 2009). Cellular processes within the photoreceptors might additionally adapt the eye.

The pupillary reflex is usually considered to be a fast mechanism of adaptation. A fast pupil response is indeed characteristic for the eyes of some cephalopod species (Table 1). It takes the pupils of *Lolliguncula brevis* (McCormick and Cohen, 2012), *Sepia officinalis*, and *Eledone cirrhosa* (Douglas et al., 2005), *Todarodes pacificus* (Matsui et al., 2016) as well as *Sepioloidea lineolate* (Douglas, 2018) only 1–3 s to constrict; the t_50_-values, defined as the time required to achieve 50% pupil constriction, were assessed as 0.3–1.5 s in these species. In contrast, the pupils of *Japetella diaphana* and *Nautilus pompilius* react more slowly to changes in light condition (Table 1; Hurley et al., 1978; Douglas, 2018). Previous studies described that diffuse light is sufficient to cause constriction of the cephalopod pupil (Beer, 1897; Weel and Thore, 1936). Most cephalopods seem to lack a consensual pupil response (Beer, 1897; Magnus, 1902; Weel and Thore, 1936; Douglas et al., 2005; McCormick and Cohen, 2012), thus if one eye is illuminated, only the pupil of this eye constricts but not the pupil of the non-illuminated eye. Nautilus (*Nautilus pompilius*), on the other hand, has a consensual pupil response (Hurley et al., 1978), meaning that both pupils constrict even if only one eye is illuminated. In the Atlantic brief squid (*Lolliguncula brevis*), the pupil of the unstimulated eye also contracts, but less so than the pupil of the stimulated eye (McCormick and Cohen, 2012).

**TABLE 1:** Overview of the results obtained in previous studies on pupillary reactions in cephalopods including the t_50_-value, the time interval after light onset, at which half-maximum constriction is reached (in s), the maximal constriction of the pupil (in % of the fully dilated pupil before light onset), the pupillary parameter (either pupil area or pupil diameter) measured during the study, and the reference.

Species	t_50_ (s)	Maximal constriction (%)	Measure of pupillary opening	References
*Eledone cirrhosa*	0.65	<3	Area	Douglas et al., 2005
*Japetella diaphana*	6.2	8	Area	Douglas, 2018; (Figure 20)
*Sepia officinalis*	0.32	<3	Area	Douglas et al., 2005
*Sepioloidea lineolate*	0.4	2	Area	Douglas, 2018 (Figure 19)
*Loliguncula brevis*	0.49-1.2	24	Area	McCormick and Cohen, 2012
*Todarodes pacificus*	1-1.5	<20	Diameter	Matsui et al., 2016 (Figure 5)
*Nautilus pompilius*	39	20 40	Vertical diameter Horizontal diameter	Hurley et al., 1978 (Figure 1A) Hurley et al., 1978 (Figure 1B)

In this study, the pupil light and dark reaction of the common octopus, *Octopus vulgaris*, was analyzed; throughout the text, we will refer to the common octopus as octopus for simplicity. The pupil of octopus is circular when the eye is in darkness and constricted to a horizontal slit in bright light (Figures 1B,C). Previous researchers have already described some aspects of the pupil/iris of octopus such as the brain centers, nerves, and muscles controlling pupillary function (Magnus, 1902; Weel and Thore, 1936; Budelmann and Young, 1984), the histological fine structure of the iris (Froesch, 1973), and the fact that the octopus always keeps its slit-shaped pupil horizontal irrespective of body position (Wells, 1960). In a similar way as in other animals (Douglas, 2018), the pupil size of octopus is not only depending on the ambient illumination but also on other factors such as arousal (Weel and Thore, 1936). According to Weel and Thore (1936), the octopus has a non-consensual pupil response thus the pupil is only constricting when the respective eye is illuminated by light but not when the contralateral eye is illuminated. This observation is consistent with the octopus often looking at objects with one of its laterally placed eyes only (Heidermanns, 1928; Muntz, 1963; Byrne et al., 2002) and showing asymmetry in eye use (Byrne et al., 2002).

Pupillary dynamics in *Octopus vulgaris*, which, according to our knowledge, have not been quantified before, were of interest as one tessera of the mosaic of vision and the visual abilities in octopus. The rate of pupillary constriction and dilation reflects the rate of light changes experienced by the animal in its daily life, and the light range within which the pupil dilates or constricts differentially is informative regarding the animal’s light environment. Besides the documentation of the pupillary dynamics, the aim of this study was to compare the pupil light and dark reaction when the eye is illuminated axially or from above (called dorsal illumination hereafter). Observations made by ourselves and others (Hess, 1909, 1910; Douglas et al., 2005; McCormick and Cohen, 2012; Mäthger et al., 2013) suggested that the horizontal and probably even more the W- or U-shaped pupils of cephalopods serve to protect the eyes from down-welling light. Consequently, dorsal compared to axial illumination should affect the octopus pupil less; a hypothesis tested in the study at hand.

## Materials and Methods

### Experimental Animal

Pupillary reactions were documented in one wild-caught (Tuscan Archipelago of the Mediterranean Sea), female adult common octopus, *Octopus vulgaris*, with a mantle length of 6.5 cm. At the Marine Science Center Rostock, Germany, it was housed solitarily in a compartment (130 × 85 × 78 cm) of a 3,000 l seawater aquarium with a substrate composed of small stones and small pieces of corals. Large stones as well as shells were provided to allow the animal to hide underneath or to construct a den. In the aquarium, salinity was kept at 32–33 g/kg, temperature was adjusted to 21–23°C, and water quality was regularly checked. After transport, the animal was adapted to the salinity and temperature of the aquarium by adding water from the holding tank dropwise to the container the animal was residing in and that contained natural ocean water from the point of capture. During the phase of adaptation, lasting several hours, the animal was continuously monitored.

A day and night cycle with 9 h daylight, 1 h dusk and dawn, and 13 h night was achieved with the help of artificial illumination (Aqua Medic Ocean Lights, Reef blue, 2× 150 W and T52×54 W, Bissendorf, Deutschland; Starlicht KOS Cut-Case 1×13L White, Herzebrock-Clarholz, Deutschland).

During the study with an experimental phase of 2 months, the octopus was fed with a mixture of northern prawn (*Pandulus borealis*), petan fish (*Osmerus eperlanus*), and saltwater mussles (*Mytilidae* sp.) 6–7 days a week *ad libitum*.

Maintenance, care, and welfare followed published recommendations (Smith et al., 2013; Fiorito et al., 2014, 2015). This study was conducted in accordance with the directive 2010/63/EU, and maintenance and the measurements (Permit No. 6712GH00113, Staatliches Amt für Umwelt und Natur Rostock, Landesamt für Landwirtschaft, Lebensmittelsicherheit und Fischerei, Mecklenburg-Vorpommern) as well as the transport (EG Verordnung 1/2005, Reg.-Nr. 082120000714) were approved by local authorities. The ARRIVE guidelines (Kilkenny et al., 2010) checklist was the basis for the preparation of this manuscript.

### Experimental Setup

The pupillary reactions of the octopus were documented with the animal residing in its home tank (Figure 1A). For the documentation of the pupil light and dark response (PLR, PDR), the octopus eye was illuminated with light emitted from a light box that was directly attached to the aquarium from outside. The light box was installed either on the side of the aquarium to illuminate the eye axially or placed on top of the aquarium to illuminate the eye from above.

The front plate of the light box was a square acrylic plate with 25 cm side length. It allowed 92% of the light to be transmitted. This plate was indirectly illuminated by the light of three 20 W lamps (mirror reflector bulb, CIL FTD/A 20W/12 V, diameter 77 mm) reflected by aluminum foil lining the inside of the box. The position of the lamps was adjusted to achieve a homogenous illumination of the front plate varying only by ± 12% across the surface on average. The light box emitted light of wavelengths between 400 and 860 nm (measured with Ocean Optics spectrometer USB 2000). Additional infrared light at 850 nm was always illuminating the scene allowing the documentation of the pupil responses with an infrared-sensitive camera even at the lowest light intensities.

The light emitted from the light box could be dimmed with a dimmer (REV Ritter GmbH, 40–300W, 230V, Typ EMD 200). Nine different light intensities ranging from 0.7 ± 0.4 cd/m^2^ to 186.1 ± 18.7 cd/m^2^ were chosen to document the pupil responses (Table 2). The luminance of the light box was measured with a luminance meter (Minolta Luminance Meter LS-110, Japan) from the distance at which the eye of the octopus had been within the aquarium during measurements, at five points on the front plate after every measurement/light period. Final luminance values (Table 2) represent averages of all measurements per light level (axial illumination *N* = 45, dorsal illumination *N* = 40). Additionally we assessed t_50_-values of the light unit for three luminances: 0.29 ± 0.017 s for 1 cd/m^2^, 0.25 ± 0.000 s for 60 cd/m^2^, and 0.142 ± 0.003 s for 150 cd/m^2^; the t_50_ value indicates the time needed to reach half maximum luminance.

**TABLE 2:** Light levels used to elicit a pupil response of *Octopus vulgaris* during axial and dorsal illumination average luminance as well as log (luminance) in cd/m^2^ ± SD) as well as the t_50_-values (in s) determined during the respective pupillary light reaction with the number of measurements (N) performed to determine the t_50_-value.

Axial illumination				Dorsal illumination			
	
Luminance (cd/m^2^)	Log (Luminance) (cd/m^2^)	t_50_ (s)	N	Luminance (cd/m^2^)	Log (Luminance) (cd/m^2^)	t_50_ (s)	N
0.7 ± 0.4	−0.150.40	0.83	6	1.00.3	00.52	1.29	5
2.2 ± 0.8	0.340.10	0.57	7	2.40.6	0.380.22	1.06	7
17.6 ± 3.9	1.20.59	0.45	9	18.33.3	1.260.51	0.66	8
24.3 ± 5.0	1.390.70	0.50	9	26.44.7	1.420.67	0.60	8
50.9 ± 8.1	1.710.91	0.54	9	59.78.1	1.780.91	0.61	8
66.1 ± 11.8	1.821.07	0.50	9	74.09.3	1.870.97	0.56	8
104.5 ± 14.5	2.021.16	0.52	9	115.910.4	2.061.02	0.54	8
125.8 ± 18.2	2.101.26	0.52	9	142.314.6	2.151.16	0.59	8
170.3 ± 21.6	2.231.33	0.49	9	186.118.7	2.271.27	0.59	8

The pupil responses were recorded with a camera (DSP CCD Camera XC229SR) at 30 fps, which was always filming the octopus eye axially (Figures 1B,C).

### Experimental Procedure

**FIGURE 2:**
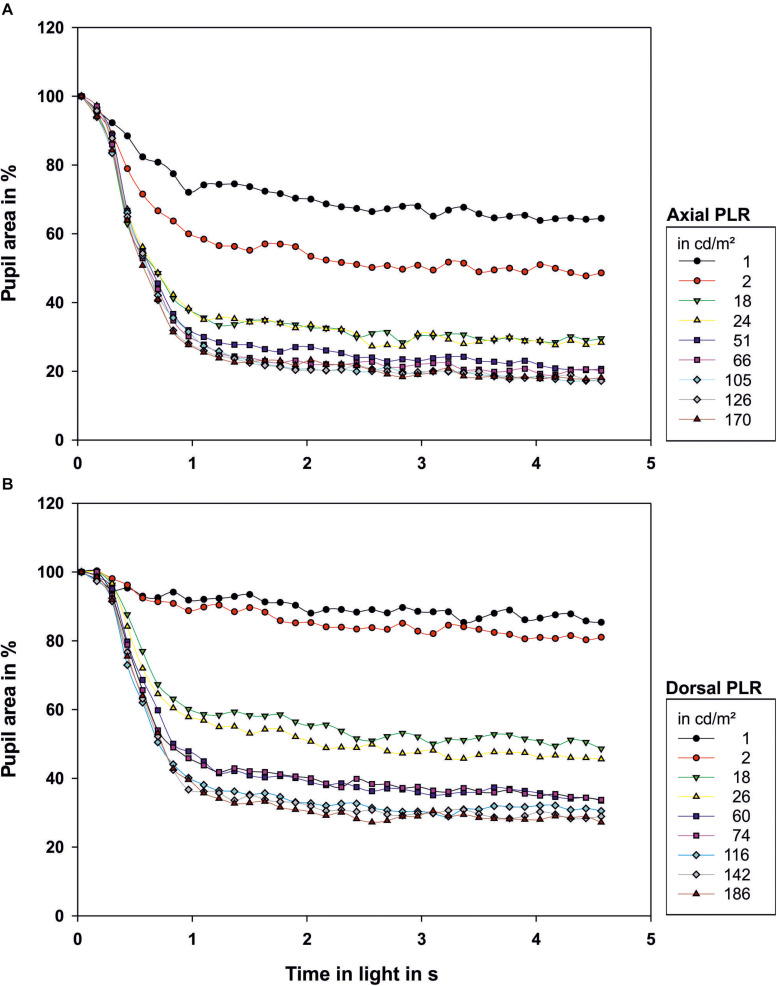
Light reaction of the octopus pupil when **(A)** illuminated axially or **(B)** from dorsal. The area of the pupil is depicted as percentage of the area of the maximally dilated pupil at light onset. Each data point represents the mean value of the pupillary area of 3–9 measurements. The luminance of the light source in cd/m^2^ measured from the distance at which the eye of the octopus had been during measurement is indicated in the legend. Light onset is zero on the time axis.

The octopus was filmed when sitting in its den with only its eyes protruding. Before each experimental session, the room was completed darkened, and the octopus was kept in darkness for a minimum of 3 min during which time the pupil dilated fully (Figure 1C). After this initial dark period (dark period 1, Figure 1E), the light source was switched on with the lowest luminance (light period with light level 1, Figure 2E and Table 2). The PLR was recorded during this light period that lasted 15–600 s depending on the experimental phase. Immediately after the recordings were finished, the luminance of the front plate was measured. Before the next measurement, the animal was again in darkness for at least 3 min, ensuring that the pupil was fully dilated before the animal was exposed to the next light level. This way the luminance was increased stepwise from light level 1 to light level 9 (Table 2).

To assess the PDR, the octopus was first exposed to a dark period (dark period 1, Figure 1F), and second to a light period (Figure 1F) as during the documentation of the PLR. After this light period, during which a specific light level was set up (Table 2), the light was switched off, and the camera recorded the response of the pupil to sudden darkness over time (dark period 2, Figure 1F).

### Data Analysis

For every PLR measurement, 140 frames (4.6 s) during the light period with the first frame at light onset were analyzed in ImageJ (Wayne Rasband). For the documentation of the PDR, 140 frames (4.6 s) during the dark period with the first frame at light turn-off were taken for the analysis, respectively. Thus the PLR and the PDR were assessed over a time interval of 4.6 s with time steps of 0.125 s.

Pupillary area (Figure 1D) was measured with ImageJ (Wayne Rasband, W.S., ImageJ, U. S. National Institutes of Health, Bethesda, MD, United States, 1997–2018^1^) on all selected frames. A few frames could not be analyzed as

(1) the frame was blurred, mainly due to movements of the animal, or
(2) the pupil was fully or partially occluded, for example by an arm of the octopus.

To present the PLR and PDR, the pupil area was expressed as percent of the fully dilated pupil area measured on the first frame of the corresponding light period (Douglas et al., 1998, 2005; McCormick and Cohen, 2012).

The following aspects were analyzed:

(1) Pupil light reaction (PLR) – analysis of the pupil over time as a reaction to axial and dorsal light.
  a. t_50_-value defined as the time after the onset of the light phase to achieve 50% maximum pupil constriction, derived from the minimal and the maximal pupil area measured.
  b. the PLR over a prolonged time period of 10 min as exemplary measurements
  c. maximum pupil constriction, defined as the minimal pupil area occurring during a measurement
(2) Pupil dark reaction (PDR) – analysis of the pupil area over time as reaction to darkness after axial and dorsal illumination in the light period.
  a. the PDR over a prolonged time period of 10 min as exemplary measurements
  b. maximum pupil dilation relative to maximal pupil area assessed right at the onset of the light period.

The data were statistically analyzed in R [R Core Team (2017) R: A language and environment for statistical computing. R Foundation for Statistical Computing, Vienna, Austria^2^ ].

## Results

**FIGURE 3:**
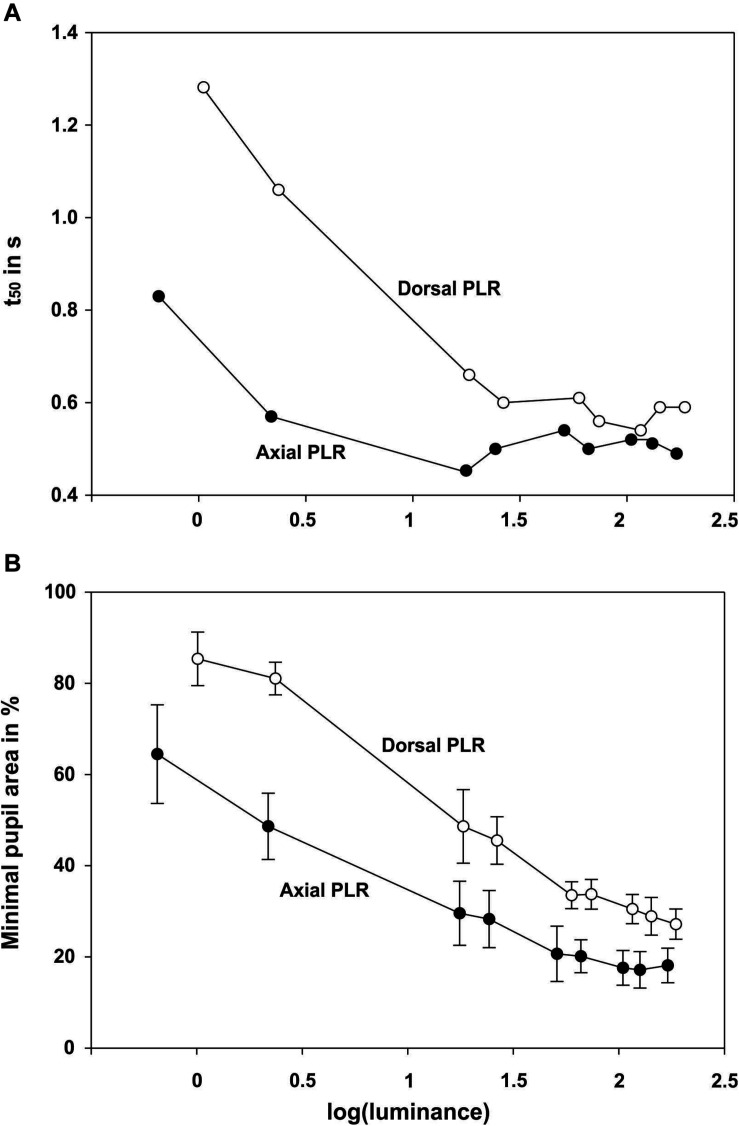
Summary graphs showing for the pupillary light reaction with dorsal and axial illumination **A** the t_50_ value (in s) and **B** minimal pupil area (in % of the maximum pupil area at light onset) as a function of log (luminance).

After light onset, the pupil constricted within less than 1 s (Figure 2; all measurements can be found in Supplementary Figure 1 and Figure 3). The t_50_-values ranged from 0.45 to 0.83 s for axial illumination and from 0.54 to 1.29 s for dorsal illumination (Table 2). The pupil response was significantly faster to axial illumination than to dorsal illumination for low luminance values up to 2.4 cd/m^2^ (general linear model with comparison of means, *p* < 0.01; Figure 3A). For higher luminance values, pupil reaction was not significantly faster during axial illumination in comparison to dorsal illumination (*p* > 0.05).

With luminance values up to 17.6 cd/m^2^ for axial illumination (general linear model with comparison of means, *p* < 0.001) and to 26.4 cd/m^2^ for dorsal illumination (general linear model with comparison of means, *p* < 0.05), the pupil only closed partially (Figures 2, 3B). Higher ambient luminance values did not result in a significantly different pupil reaction.

**FIGURE 4:**
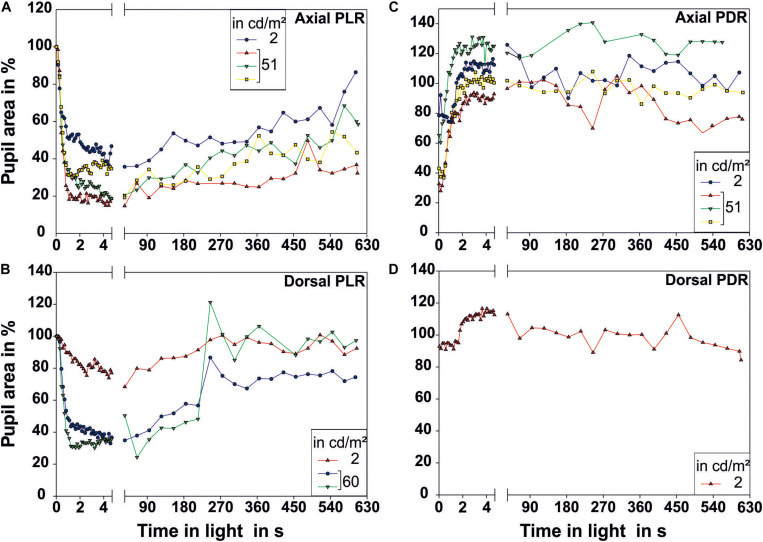
Pupillary reactions measured over a prolonged time period of 10 min. **(A,B)** Illustrate the pupillary light reaction (PLR) for **(A)** axial (*N* = 4) and **(B)** dorsal illumination (*N* = 3). **(C,D)** Illustrate the pupillary dark reaction (PDR) for **(C)** axial (*N* = 4) and **(D)** dorsal illumination (*N* = 1). Only one low and one higher luminance were chosen for these long-term recordings. All measurements performed are plotted in this figure. The pupil size on the first frame of the light period was taken as 100% pupillary area. Each data point represents one measurement of pupillary area. The luminance of the light source in cd/m^2^ measured from the distance at which the eye of the octopus had been during measurement is indicated in the legend. Light onset is zero on the time axis.

Single measurements over a time period of 10 min revealed that the pupil finally re-dilated in light up to 86.4% of its maximal area for axial illumination (Figure 4). For dorsal illumination, the pupil even dilated completely with the pupil area reaching values above 100% of its initial maximal area at the onset of the light period.

The pupil area at maximum constriction of 10.3% of the dilated pupil area for axial illumination and 18.1% for dorsal illumination was reached after 4.3 and 3.5 s, respectively. For every light level, the pupil area was smaller during axial than during dorsal illumination (general linear model with comparison of means, *p* < 0.05).

**FIGURE 5:**
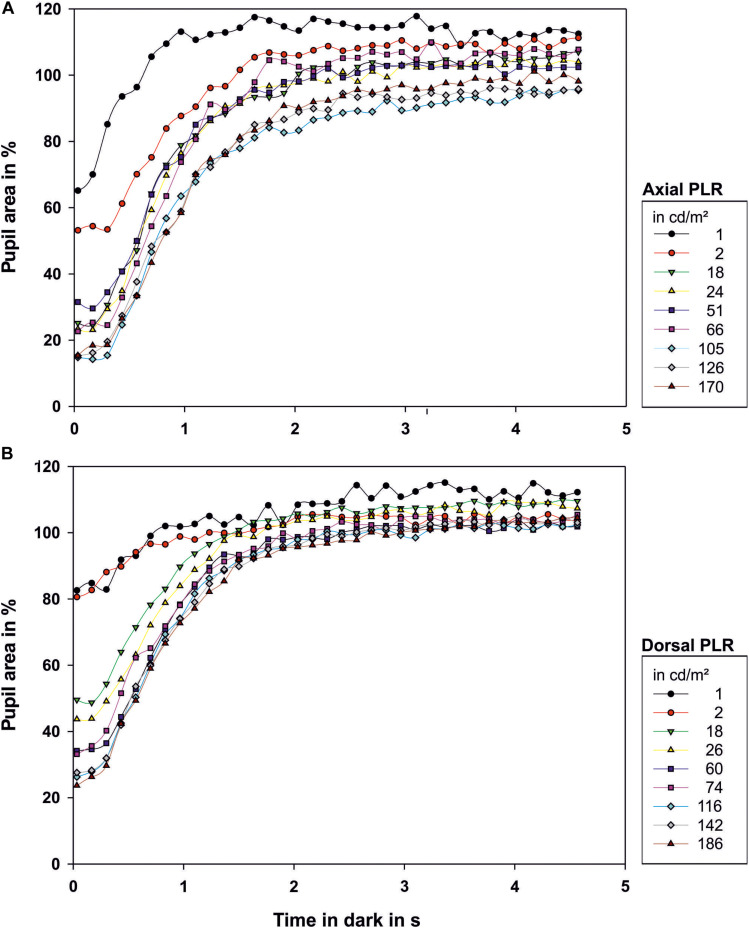
Dark reaction of the octopus pupil when **(A)** illuminated axially or **(B)** from dorsal. The pupil size on the first frame of the light period was taken as 100% pupillary area. Therefore values above 100% occur. Zero on the time axis indicates when the light was switched off. Each data point represents the mean value of the pupillary area of 3–9 measurements. The luminance of the light source in cd/m^2^ in the light period preceding the respective dark phase 2 is indicated in the legend.

During the PDR, full pupillary dilation (Figure 5; all measurements can be found in Supplementary Figure 2 and Figure 4) took slightly longer than 1 s especially when the eye was illuminated axially. Under this condition, there was more variation in the final pupil area than under dorsal illumination, and low light levels experienced before the documentation of the PDR caused the pupil to dilate to a larger area than the pupil area measured on the first frame of the light period. Prolonged measurements in darkness revealed that pupillary area varied drastically even in darkness with the pupil sometimes constricting even down to 66.9% for previous axial illumination and 84.3% for previous dorsal illumination (Figure 3).

## Discussion

In this study, we documented the course of the pupil light and dark reaction of one common octopus, *Octopus vulgaris*. This octopus showed vivid pupillary responses, and the resulting data are similar to pupillary reactions documented for other animals including other cephalopods (Douglas, 2018). Furthermore the reproducibility of our measurements is high. Thus we are confident that our measurements are reliable for this individual. Future measurements could help to clarify whether our results are representative for the species by measuring pupillary responses in other octopus individuals that will allow us to document their pupillary response without restrain. At the same time, the non-consensual pupillary response of the octopus described by Weel and Thore (1936) could be quantified in these future experiments.

Only weak light of <1 cd/m^2^ was necessary to cause the pupil of *Octopus vulgaris* to constrict, similar to observations by previous researchers (Beer, 1897; Weel and Thore, 1936). A pupil constriction upon experiencing low ambient light is generally found in animals that are active under low light conditions (Douglas, 2018). The octopus experiences low light intensities for example in its den or when being active at night (Woods, 1965; Altman, 1966; Kayes, 1974) or during dusk or dawn (Mather, 1988). Thus a pupillary response adapted to operate under dim light conditions fits the ecology of the common octopus.

After the light was switched on, the octopus pupil closed within approximately 1 s. It needs to be mentioned that, in this study, we might have slightly overestimated the t_50_ value as the light was not instantaneously on; in the future, the pupillary responses could be re-determined and compared with new data obtained with a different light source that can be switched on instantaneously. The PLR of octopus as currently determined was slower than that of birds, but similar to that of humans or teleost fish (for overview see Douglas, 2018). Time for pupil closure was also in the same range as for many previously examined cephalopods (Table 1; Douglas et al., 2005; McCormick and Cohen, 2012; Matsui et al., 2016; Douglas, 2018). Most likely, the PLR is fast in these cephalopods as they experience rapid changes in ambient illumination in their natural habitat. *Octopus vulgaris* that can show diurnal activity (Meisel et al., 2003, 2006), might experience a fast and large increase of light intensity when leaving its dark den in shallow water during daytime. In this or similar situations, light incidence into the eye is regulated rapidly by the pupil, avoiding photon overload. However, under these circumstances, the pupil alone is not sufficient to adapt the eye; it has to be accompanied by other mechanisms (Douglas et al., 2005) such as pigment migration and/or changes in the length of the photoreceptors as mentioned in the introduction.

During our measurements, light caused the pupil to maximally constrict to approximately 10% of the dark-adapted pupil area. This maximal value is similar to previous results obtained in cephalopods (Table 1). However, we never observed the octopus pupil to constrict even further allowing light to only penetrate the eye at the two corners of the pupil as described for *Octopus vulgaris* by Weel and Thore (1936) and by Heidermanns (1928). It remains to be determined whether the pupil of other *Octopus vulgaris* individuals would constrict to less than 10% of the dark-adapted pupil area. A constriction down to 10% fits to the octopus being a shallow-water species (Jereb et al., 2014; Sanchez et al., 2015). By contrast, many species diving fast and deep can close the pupil to much smaller areas (see for example data obtained in seals Levenson and Schusterman, 1997), which prepares the eyes for the darkness encountered at depth.

Under dorsal illumination, light had less effect on pupil area, compared to axial illumination; higher light intensities were required to cause pupillary constriction, and maximal constriction was 18%, thus less closed by almost a factor of two. In line with Jagger and Sands (1999) and Mäthger et al. (2013), we conclude that these effects were likely caused by the horizontal pupillary slit of octopus shielding light from above. Such a shadow effect is beneficial in the habitat of octopus (Jereb et al., 2014), in which light incidence is almost exclusively from above (see for example Figure 5B in Mäthger et al., 2013). As a consequence, a homogeneous illumination in the eye is most likely achieved (Jagger and Sands, 1999; Mäthger et al., 2013), and local adaptation of the retina will not be necessary. To support this consideration, a retinal illumination map would need to be computed for octopus the same way as was done in *Sepia officinalis* (Mäthger et al., 2013) and the octopus pupillary responses would need to be recorded with illumination from different sectors for comparison.

The PLR was slightly faster than the PDR in octopus as documented for humans (Mathôt, 2018) or insects (Stavenga, 1979; Stavenga et al., 1979). The fact that both reactions are fast, suggests that the eye of octopus possesses a dilator and a sphincter muscle. Froesch (1973) described a muscle layer within the iris of octopus, however, he could not distinguish between sphincters and dilators; an aspect that still awaits examination in a future project. Modeling the pupillary responses of *Octopus vulgaris* (see models for the human pupil such as Longtin and Milton, 1989; Pamplona et al., 2009; Fan and Yao, 2011; Johansson and Balkenius, 2017), another possible direction of current research, might additionally help to understand the underlying mechanisms.

We observed variations in pupil area under all experimental conditions. These variations can possibly be explained by pupil size serving additional functions besides the regulation of light incidence. First, if the lens suffers from longitudinal spherical aberration, a constricted pupil can result in an enhanced quality of the image, because light is restricted to the central part of the lens (Sivak, 1991; Douglas et al., 2005). Most likely the spherical lenses of *Octopus vulgaris* and other octopus species are corrected for longitudinal spherical aberration (Jagger and Sands, 1999). However, if some residual longitudinal spherical aberration was present as in other cephalopods (Sivak, 1982; Sroczynski and Muntz, 1985; Sroczynski and Muntz, 1987; Sivak, 1991; Sivak et al., 1994; Kröger and Gislen, 2004; Sweeney et al., 2007), closing the aperture of the eye would benefit image quality. Second, depth of focus is large in an eye with constricted pupil; being horizontally slit-shaped, depth of field is increased for horizontal contours (Banks et al., 2015). At specific times, it might be advantageous to have several objects in focus simultaneously eliminating the need for strong accommodation, even though *Octopus vulgaris* might be able to accommodate (Beer, 1897). Third, a constricted pupil could help to camouflage the eye. Cephalopods are masters of camouflage, however, camouflaging the eye is challenging especially if the pupil is dilated and can thus be seen as a large, dark, and regular spot. In contrast, a constricted pupil is less conspicuous than a dilated pupil as the dark area is smaller. Thus constricting the pupil, in combination with chromatophores and iridophores on the iris (Froesch, 1973), might conceal the eye for example in the presence of predators. In a benthic animal, predators might primarily approach from above, the direction which might be perfectly shielded by the horizontal arrangement of the pupil of octopus if the dorsal part of the eye lid indeed serves as a dorsal shield; an aspect that needs to be worked on in the future. An eye concealment function of the pupil has already been put forward for bottom-dwelling fish with mobile irises (Douglas, 2018; Youn et al., 2019) that stand in contrast to most teleost fish with immobile irises (Douglas, 2018). Lastely, a constricted and especially constricted off-axis pupil can increase chromatic blur of the optical system which monochromats might be able to use to obtain color information nevertheless (Stubbs and Stubbs, 2016).

While the previously mentioned functions relate to a constricted pupil, a dilated pupil can also fulfill additional functions; these might explain why we observed a strong re-dilation in light over time. The pupil might dilate due to arousal (Weel and Thore, 1936), or the dilated pupil could serve as an intraspecific signal (Packard, 1972). Additionally, if the pupil dilated when viewing close objects, defined as the near response, then the animal could use accommodation to judge its distance to an object (Wells, 1966). In general, pupil dilation can also be part of a deimatic display, as it possibly creates the illusion of being larger which could be essential when a predator suddenly appears (Wells, 1966; Hanlon and Messenger, 1988, 1996; Messenger, 2001).

## Conclusion

In conclusion, the common octopus can rely on a mobile pupil to assist light and dark adaptation in its at times light-inhomogeneous environment.

## Data Availability Statement

All datasets generated for this study are included in the article/Supplementary Material.

## Ethics Statement

The animal study was reviewed and approved by the Staatliches Amt für Umwelt und Natur Rostock, Landesamt für Landwirtschaft Lebensmittelsicherheit und Fischerei, Mecklenburg-Vorpommern.

## Author Contributions

CS and FH performed the measurements and analyzed the data. All authors conceived the experiment and contributed to and approved the final version of the manuscript.

## Conflict of Interest

The authors declare that the research was conducted in the absence of any commercial or financial relationships that could be construed as a potential conflict of interest.

## Footnotes

1 https://imagej.nih.gov/ij/

2 https://www.R-project.org/

## References

[B1] Altman J. S. (1966). *The Behaviours of Octopus Vulgaris Lam. in its Natural Habitat: A Pilot Study.* Sliema: Underwater Association of Malta.

[B2] Babuchin A. (1864). Vergleichend histologische Studien - übder den Bau der Cephalopodenretina. *Würzburger Naturwissenschaftliche Zeitschrift* 5 127–140.

[B3] Banks M. S. Sprague W. W. Schmoll J. Parnell J. A. Q. Love G. D. (2015). Why do animal eyes have pupils of different shapes? *Sci. Adv.* 1:e1500391. 10.1126/sciadv.1500391 26601232 PMC4643806

[B4] Beer T. (1897). Die Accommodation des kephalopodenauges. *Pflüger’s Arch. Physiol.* 67 541–587. 10.1007/bf01661630

[B5] Bozzano A. Pankhurst P. M. Moltschaniwsky N. A. Villaneuva R. (2009). Eye development in southern calamary, *Sepioteuthis australis*, embryos and hatchlings. *Mar. Biol.* 156 1359–1373. 10.1007/s00227-009-1177-2

[B6] Budelmann B. U. (1994). Cephalopod sense organs, nerves and the brain: adaptations for high performance and lifestyle. *Mar. Freshw. Behav. Physiol.* 25 13–33. 10.1080/10236249409378905

[B7] Budelmann B. U. (1996). Active marine predators: the sensory world of cephalopods. *Mar. Freshw. Behav. Physiol.* 27 59–75. 10.1080/10236249609378955

[B8] Budelmann B. U. Young J. Z. (1984). The statocyst-oculomotor system of *Octopus vulgaris*: extraocular eye muscles, eye muscle nerves, statocyst nerves and the oculomotor centre in the central nervous system. *Philos. Trans. R. Soc. Biol. Charact.* 306 159–189. 10.1098/rstb.1984.0084

[B9] Byrne R. A. Kuba M. Griebel U. (2002). Lateral asymmetry of eye use in *Octopus vulgaris*. *Anim. Behav.* 64 461–468. 10.1006/anbe.2002.3089

[B10] Daw N. W. Pearlman A. L. (1974). Pigment migration and adaptation in the eye of the squid, *Loligo pealei*. *J. Gen. Physiol.* 63 22–36. 10.1085/jgp.63.1.22 4810208 PMC2203541

[B11] Douglas R. H. (2018). The pupillary light response of animals; a review of their distribution, dynamics, mechanisms and functions. *Prog. Retin. Eye Res.* 66 17–48. 10.1016/j.preteyeres.2018.04.005 29723580

[B12] Douglas R. H. Harper R. D. Case J. F. (1998). The pupil of a teleost fish, *Porichthys notatus*: description and comparison to other species. *Vis. Res.* 38 2697–2710. 10.1016/s0042-6989(98)00021-2 9775319

[B13] Douglas R. H. Williamson R. Wagner H.-J. (2005). The pupillary response of cephalopods. *J. Exp. Biol.* 208 261–265. 10.1242/jeb.01395 15634845

[B14] Fan X. Yao G. (2011). Modeling transient pupillary light reflex induced by a short light flash. *IEEE Trans. Biomed. Eng.* 58 36–42. 10.1109/tbme.2010.2080678 20876003 PMC4318650

[B15] Fiorito G. Affuso A. Anderson D. B. Basil J. A. Bonnaud L. Botta G. (2014). Cephalopods in neuroscience: regulations, research and the 3Rs. *Invertebr. Neurosci.* 14 13–36. 10.1007/s10158-013-0165-x 24385049 PMC3938841

[B16] Fiorito G. Affuso A. Basil J. A. Cole A. de Girolamo P. D’Angelo L. (2015). Guidelines for the care and welfare of cephalopods in research – a consensus based on an initiative by CephRes, FELASA and the Boyd Group. *Lab. Anim.* 49 1–90. 10.1177/0023677215580006 26354955

[B17] Froesch D. (1973). On the fine structure of the *Octopus iris*. *Z. Zellforschung* 145 119–129. 10.1007/bf00307193 4360461

[B18] Gleadall I. G. Ohtsu K. Gleadall E. Tsukahara Y. (1993). Screening pigment migration in the *Octopus* retina includes control by dopaminergic efferents. *J. Exp. Biol.* 185 1–16.

[B19] Glockauer A. (1915). Zur Anatomie und Histologie des Cephalopodenauges. *Z. Wissenschaftliche Zool.* 113 325–360.

[B20] Hagins W. A. Liebman P. A. (1962). Light-induced pigment migration in the squid retina. *Biol. Bull.* 123:498.

[B21] Hanlon R. T. Messenger J. B. (1988). Adaptive coloration in young cuttlefish (*Sepia officinalis* L.): the morphology and development of body patterns and their relation to behaviour. *Philos. Trans. R. Soc. Biol. Charact.* 320 437–487. 10.1098/rstb.1988.0087

[B22] Hanlon R. T. Messenger J. B. (1996). *Cephalopod Behaviour.* Cambridge: Cambridge University Press.

[B23] Heidermanns C. (1928). Messende Untersuchungen über das Formensehen der Cephalopoden und ihre optische Orientierung im Raume. *Zool. Jahrbuecher Abteilung Allgemeine Zool. Physiol.* 45 346–349.

[B24] Hess C. (1905). Beiträge zur Physiologie und Anatomie des Cephalopodenauges. *Pflügers Arch. Gesamte Physiol.* 109 393–439. 10.1007/bf01677979

[B25] Hess C. (1909). Die Accommodation der Cephalopoden. *Arch. Augenheilkunde* 64 125–152.

[B26] Hess C. (1910). Untersuchungen über den Lichtsinn bei wirbellosen Tieren. *Pflüger Arch. Gesamte Physiol.* 136 282–367. 10.1007/bf01681999

[B27] Hesse R. (1900). Untersuchungen über die Organe der Lichtempfindung bei niederen Tieren. Die Retina der Cephalopoden. *Z. Wissenschaftliche Zool.* 68 379–477. 10.1007/bf00339022

[B28] Hurley A. C. Lange G. D. Hartline P. H. (1978). The adjustable “pinhole camera” eye of Nautilus. *J. Exp. Zool.* 205 37–44. 10.1002/jez.1402050106

[B29] Jagger W. S. Sands P. J. (1999). A wide-angle gradient index optical model of the crystalline lens and eye of the *Octopus*. *Vis. Res.* 39 2841–2852. 10.1016/s0042-6989(99)00012-7 10492814

[B30] Jereb P. Roper C. F. E. Norman M. D. Finn J. K. (2014). *Cephalopods of the World - An Annotated and Illustrated Catalogue of Cephalopod Species Known to Date. Volume 3. Octopods and Vampire Squids.* Rome: FAO.

[B31] Johansson B. Balkenius C. (2017). A computational model of pupil dilation. *Connect. Sci.* 30 1–15. 10.1080/09540091.2016.1271401

[B32] Kayes R. J. (1974). The daily activity pattern of *Octopus vulgaris* in a natural habitat. *Mar. Behav. Physiol.* 2 337–343. 10.1080/10236247309386935

[B33] Kilkenny C. Browne W. J. Cuthill I. C. Emerson W. Altmann D. G. (2010). Improving bioscience research reporting: the ARRIVE guidelines for reporting animal research. *PLoS Biol.* 8:e1000412. 10.1371/journal.pbio.1000412 20613859 PMC2893951

[B34] Kröger R. H. H. Gislen A. (2004). Compensation for longitudinal chromatic aberration in the eye of the firefly squid, *Watasenia scintillans*. *Vis. Res.* 44 2129–2134. 10.1016/j.visres.2004.04.004 15183679

[B35] Land M. F. (1984). “Molluscs,” in *Photoreception and Vision in Invertebrates*, ed. Ali M. A. (New York, NY: Plenum Press), 699–725. 10.1007/978-1-4613-2743-1_20

[B36] Levenson D. H. Schusterman R. J. (1997). Pupillometry in seals and sea lions: ecological implications. *Can. J. Zool.* 75 2050–2057. 10.1139/z97-838

[B37] Longtin A. Milton J. G. (1989). Modelling autonomous oscillations in the human pupil light reflex using non-linear delay-differential equations. *Bull. Math. Biol.* 51 605–624. 10.1016/s0092-8240(89)80103-x 2804468

[B38] Magnus R. (1902). Die Pupillarreaction der Octopoden. *E. Pflüger Arch. Physiol.* 92 623–643. 10.1007/bf01790186

[B39] Mather J. A. (1988). Daytime activity of juvenile *Octopus vulgaris* in Bermuda. *Malacologia* 29 69–76.

[B40] Mäthger L. M. Hanlon R. T. Hakansson J. Nilsson D.-E. (2013). The W-shaped pupil in cuttlefish (*Sepia officinalis*): functions for improving horizontal vision. *Vis. Res.* 83 19–24. 10.1016/j.visres.2013.02.016 23474299

[B41] Mathôt S. (2018). Pupillometry:psychology, physiology, and function. *J. Cogn.* 1 1–23. 10.5334/joc.18 31517190 PMC6634360

[B42] Matsui H. Takayama G. Sakurai Y. (2016). Physiological response of the eye to different colored light-emitting diodes in Japanese flying squid *Todarodes pacificus*. *Fish. Sci.* 82 303–309. 10.1007/s12562-015-0965-5

[B43] McCormick L. R. Cohen J. H. (2012). Pupil light reflex in the Atlantic brief squid, *Lolliguncula brevis*. *J. Exp. Biol.* 215 2677–2683. 10.1242/jeb.068510 22786645

[B44] Meisel D. V. Byrne M. Kuba M. Mather J. A. Ploberger W. Reschenhofer E. (2006). Contrasting activity patterns of two related *Octopus* species, *Octopus macropus* and *Octopus vulgaris*. *J. Comp. Psychol.* 120 191–197. 10.1037/0735-7036.120.3.191 16893256

[B45] Meisel D. V. Byrne R. A. Kuba M. Griebel U. Mather J. A. (2003). “Circadian rhythms in *Octopus vulgaris*,” in *Coleoid Cephalopods Through Time*, eds Warnke K. Keupp H. Boletzky S. V. (Berlin: Berlin Paläobiologische Abhandlung), 171–177.

[B46] Messenger J. B. (1979). The eyes and skin of *Octopus*: compensating for sensory deficiencies. *Endeavour* 3 92–98. 10.1016/0160-9327(79)90096-6

[B47] Messenger J. B. (2001). Cephalopod chromatophores: neurobiology and natural history. *Biol. Rev.* 76 473–528. 10.1017/s1464793101005772 11762491

[B48] Muntz W. R. A. (1963). Intraretinal transfer and the function of the optic lobes in *Octopus*. *Q. J. Exp. Psychol.* 15 116–124. 10.1080/17470216308416562

[B49] Muntz W. R. A. (1977). Pupillary response of cephalopods. *Symp. Zool. Soc. Lond.* 38 277–285.

[B50] Muntz W. R. A. Ray U. (1984). On the visual system of *Nautilus pompilius*. *J. Exp. Biol.* 109 253–263.

[B51] Packard A. (1972). Cephalopods and fish: the limits of convergence. *Biol. Rev.* 47 241–307. 10.1111/j.1469-185x.1972.tb00975.x

[B52] Pamplona V. F. Oliveira M. M. Baranoski G. G. (2009). Photorealtistic models for pupil light reflex and iridal pattern deformation. *ACM Trans. Graph.* 28 106.101–106.112.

[B53] Rawitz B. (1891). Zur Physiologie der Cephalopodenretina. *Arch. Anat. Physiol.* 5/6 367–372.

[B54] Sanchez P. Villanueva R. Jereb P. Guerra A. Gonzalez A. F. Sobrino I. (2015). “Octopus,” in *Cephalopod biology and fisheries in Europe: II. Species accounts*, eds Jereb P. Allcock L. Lefkaditou E. Piatkowski U. Hastie L. C. Pierce G. J. (Copenhagen: ICES International Council for the Exploration of the Sea).

[B55] Schaeffel F. Murphy C. J. Howland H. C. (1999). Accommodation in the cuttlefish (*Sepia officinalis*). *J. Exp. Biol.* 202 3127–3134. 10539961 10.1242/jeb.202.22.3127

[B56] Sivak J. G. (1982). Optical properties of a cephalopod eye (the short finned squid, *Illex illecebrosus*). *J. Comp. Physiol. A* 147 323–327. 10.1007/bf00609666

[B57] Sivak J. G. (1991). Shape and focal properties of the cephalopod ocular lens. *Can. J. Zool.* 69 2501–2506. 10.1139/z91-354

[B58] Sivak J. G. West J. A. Campbell M. C. (1994). Growth and optical development of the ocular lens of the squid (*Sepioteuthis lessoniana*). *Vis. Res.* 34 2177–2187. 10.1016/0042-6989(94)90100-7 7941414

[B59] Smith J. A. Andrews P. L. R. Hawkins P. Louhimies S. Ponte G. Dickel L. (2013). Cephalopod research and EU Directive 2010/63/EU: requirements, impacts and ethical review. *J. Exp. Mar. Biol. Ecol.* 447 31–45. 10.1016/j.jembe.2013.02.009

[B60] Sroczynski S. Muntz W. R. A. (1985). Image structure in *Eledone cirrhosa*, an *Octopus*. *Zool. Jahrbücher Abteilung Allgemeine Zool. Physiol.* 89 157–168.

[B61] Sroczynski S. Muntz W. R. A. (1987). The optics of oblique beams in the eye of *Eledone cirrhosa*, an *Octopus*. *Zool. Jahrbücher Abteilung Allgemeine Zool. Physiol.* 91 419–446.

[B62] Stavenga D. G. (1979). Visual pigment processes and prolonged pupillary responses in intact photoreceptor cells. *Biophys. Struct. Mech.* 5 175–185. 10.1007/bf00535446 22730591

[B63] Stavenga D. G. Bernard G. D. Chappell R. L. Wilson M. (1979). Insect pupil mechanisms I. On the pigment migration in the retinula cells of Hymenoptera (suborder Apocrita). *J. Comp. Physiol.* 129 199–205. 10.1007/bf00657654

[B64] Stubbs A. L. Stubbs C. W. (2016). Spectral discrimination in color blind animals via chromatic aberration and pupil shape. *PNAS* 113 8206–8211. 10.1073/pnas.1524578113 27382180 PMC4961147

[B65] Suzuki T. Inada H. Takahashi H. (1985). Retinal adaptation of Japanese common squid (*Tedarodes pacificus* Steenstrup) to light changes. *Bull. Faculty Fish. Hokkaido Univ.* 36 191–199.

[B66] Suzuki T. Takahashi H. (1988). Responses of the retina of flying squid *Sthenotiuthis oualaniensis* (Lesson) to light changes. *Bull. Fac. Fish Hokkaido Univ.* 39 21–26.

[B67] Sweeney A. M. Des Marais D. L. Ban Y.-E. A. Johnson S. (2007). Evolution of graded refractive index in squid lenses. *J. R. Soc. Interface* 4 685–698. 10.1098/rsif.2006.0210 17293312 PMC2373386

[B68] Weel P. B. Thore S. (1936). Über die Pupillarreaktion von *Octopus vulgaris*. *Z. Vergleichende Physiol.* 23 26–33.

[B69] Wells M. J. (1960). Proprioception and visual discrimination of orientation in *Octopus*. *J. Exp. Biol.* 37 489–499.

[B70] Wells M. J. (1966). “Cephalopod sense organs,” in *Physiology of Mollusca*, eds Wilbur K. M. Yonge C. M. (New York, NY: Academic Press), 523–545. 10.1016/b978-1-4832-3242-3.50020-3

[B71] Wiley A. (1902). *Contribution to the Natural History of the Pearly Nautilus.* Cambridge, MA: Cambridge University Press.

[B72] Woods J. (1965). *Octopus*-watching off Capri. *Animals* 7 324–327.

[B73] Youn S. Okinaka C. Mäthger L. M. (2019). Elaborate pupils in skates may help camouflage the eye. *J. Exp. Biol.* 222:jeb195966. 10.1242/jeb.195966 30665973

[B74] Young J. Z. (1963). Light- and dark-adaptation in the eyes of some cephalopods. *Proc. Zool. Soc. Lond.* 140 255–272. 10.1111/j.1469-7998.1963.tb01863.x

