# Supplementary figures and images for: The Pupillary Response of the Common Octopus (Octopus vulgaris)
Source: Front Physiol. 2020 Sep 18;11:1112. doi: 10.3389/fphys.2020.01112 (PMC7530272; doi:10.3389/fphys.2020.01112)

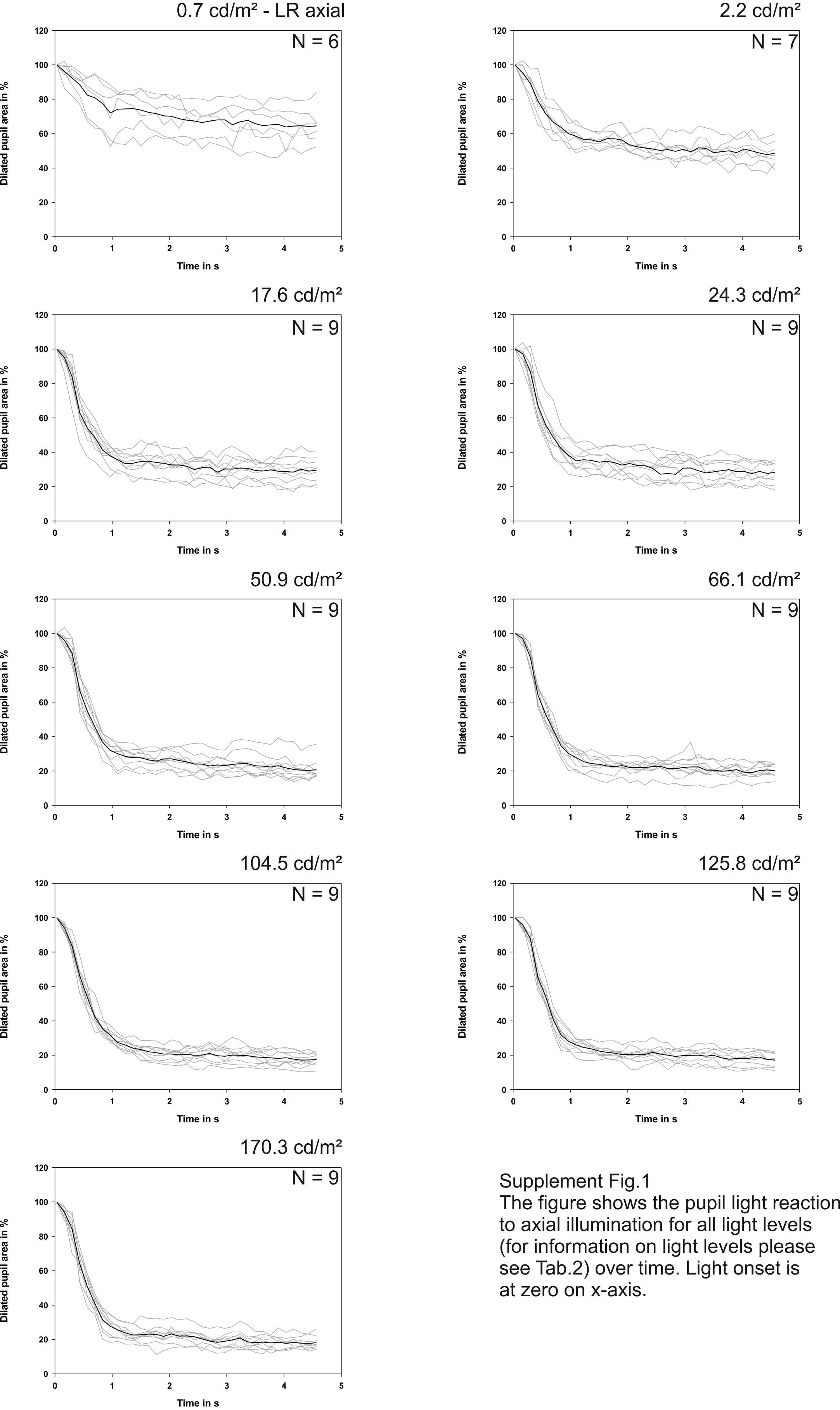

Supplement: Supplementary file 1 [file Image_1.JPEG]

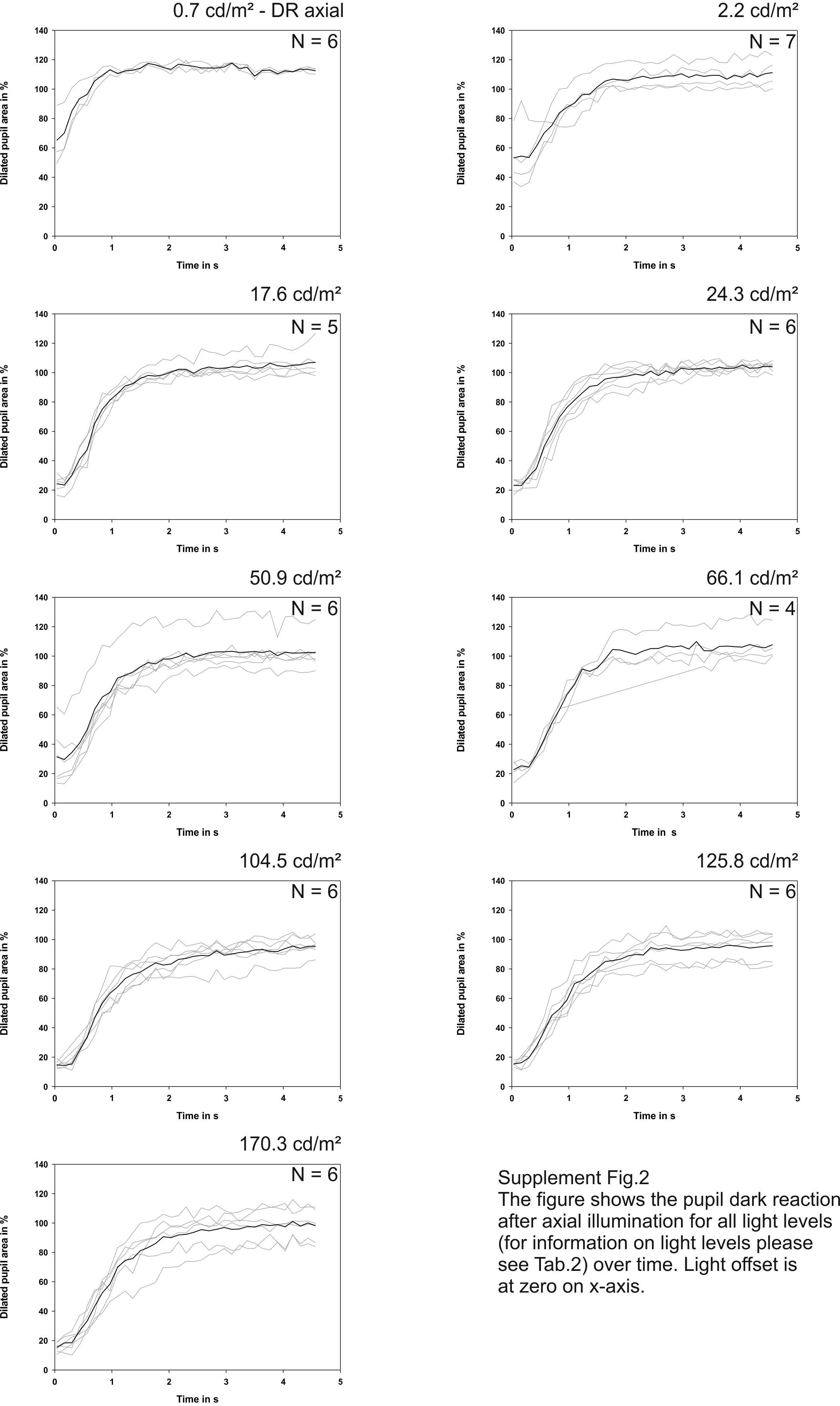

Supplement: Supplementary file 2 [file Image_2.JPEG]

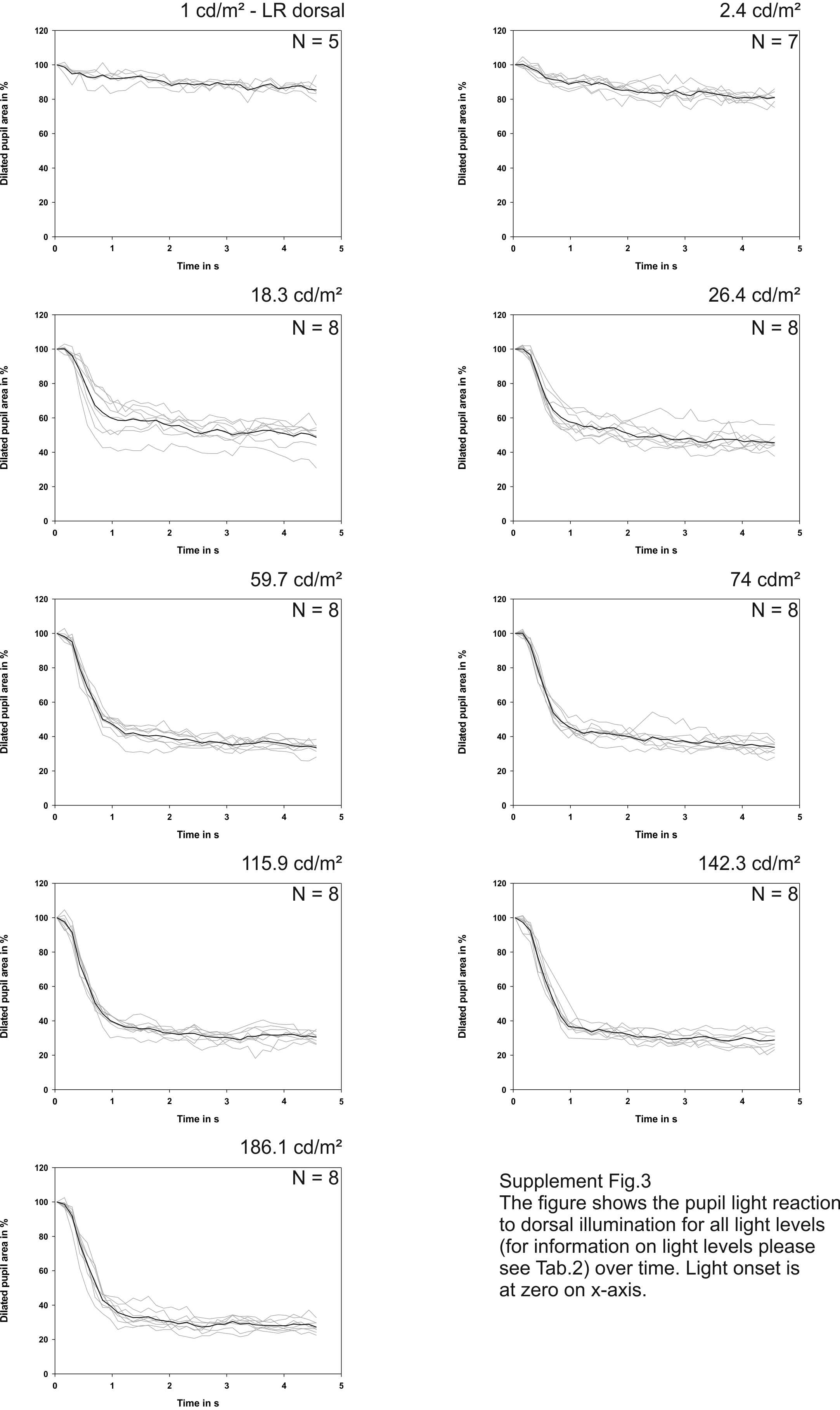

Supplement: Supplementary file 3 [file Image_3.JPEG]

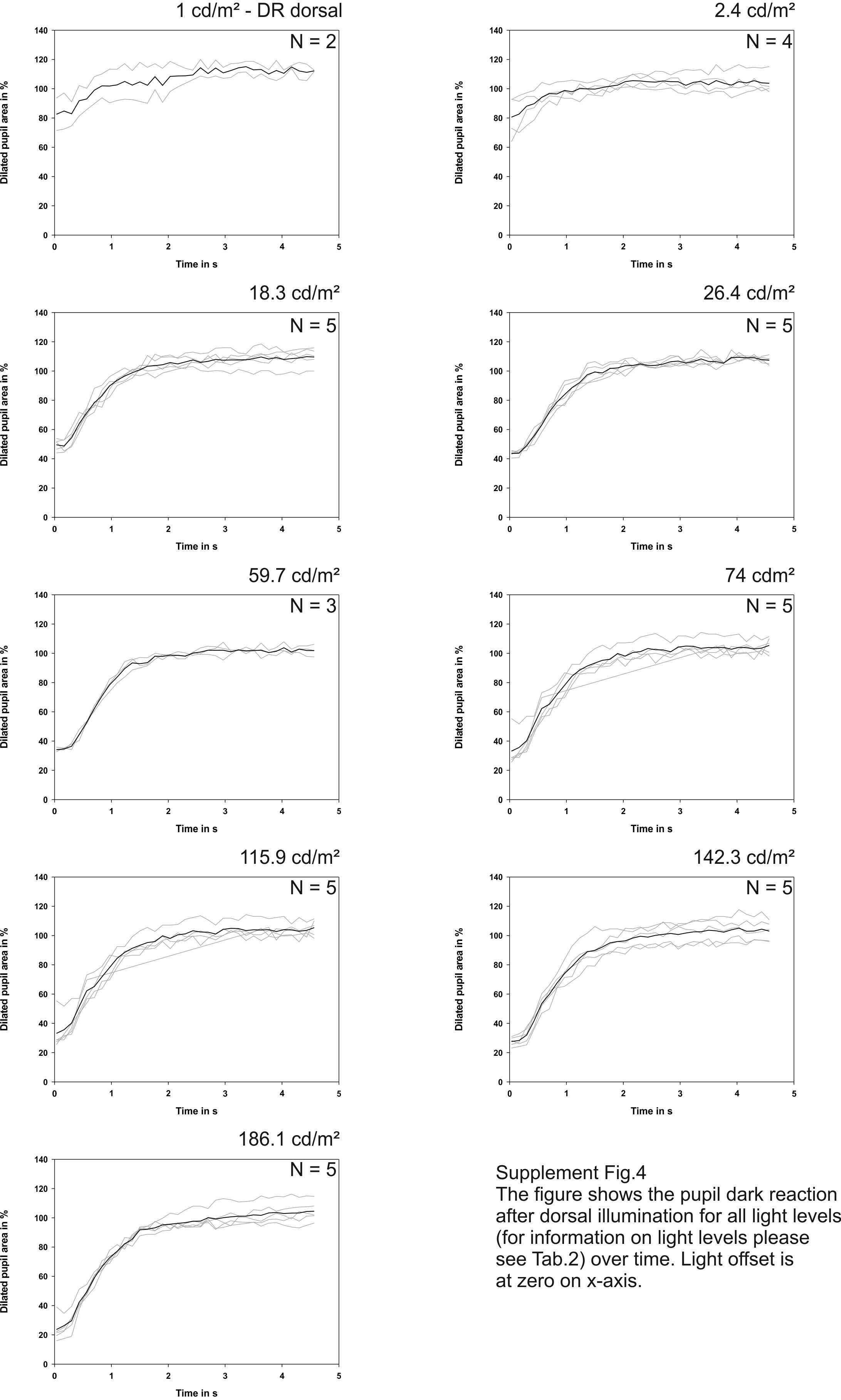

Supplement: Supplementary file 4 [file Image_4.JPEG]
